# Research Progress in the Detoxification and Resource Utilization of Chromium Slag: Recovery Technologies, Large-Scale Utilization, and Emerging Challenges—A Review

**DOI:** 10.3390/ma19102054

**Published:** 2026-05-14

**Authors:** Bin Wang, Jianjun Gao, Feng Wang, Yue Yu, Yuanhong Qi

**Affiliations:** 1 Beijing Gangyan Hydrogenmetallurgy Technology Research Institute Co., Ltd., Beijing 100081, China; 2 Hydrogen Metallurgy Center, China Iron and Steel Research Institute Group, Beijing 100081, China; 3 State Key Laboratory for Advanced Iron and Steel Processes and Products, Central Iron and Steel Research Institute Co., Ltd., Beijing 100081, China

**Keywords:** chromium slag, hexavalent chromium, detoxification, resource utilization, pyrometallurgical recovery, hydrometallurgy, solidification/stabilization (S/S), construction-material utilization, metallurgical co-treatment, long-term stability, scale-up, evidence chain

## Abstract

Chromium slag, a chromium-bearing solid waste characterized by substantial environmental hazards yet with appreciable resource potential, has become a focal topic in solid-waste pollution control and the circular economy. Centered on the overarching logic of “evidence chain–system boundary–scalable and verifiable acceptance,” this review systematically synthesizes recovery technologies, industrial-scale utilization pathways, and the key challenges associated with the detoxification and resource utilization of chromium slag. From the perspective of recovery technologies, we examine pyrometallurgical and hydrometallurgical routes, solidification/stabilization (S/S), and bioelectrochemical coupling approaches, elucidating their fundamental principles, applicability boundaries, and critical nodes where environmental burdens may be transferred across media. We emphasize that process design should concurrently consider detoxification efficiency, resource recovery performance, and whole-process pollution control. Regarding utilization pathways, this review highlights three major routes with strong scale-up relevance—metallurgical process co-treatment (CAP–sintering–blast furnace), bulk utilization in construction materials, and high-value utilization—and analyzes their industrial potential and engineering constraints. Particular attention is given to the lack of long-term leaching and durability evidence, which represents a central bottleneck limiting product-side credibility. Furthermore, we discuss cross-cutting challenges including the long-term stabilization of Cr(VI), the verifiability of “green utilization” concepts, cost and economic feasibility, and standardized acceptance criteria. We propose that future research should shift from single-process optimization toward multi-objective, system-level evaluation, and establish a full-chain evidence system covering “speciation/mineral phases–process mechanisms–environmental behavior–risk assessment–engineering scale-up–standardized acceptance.” This review aims to provide a systematic analytical framework and practical reference for improving comparability across resource-utilization technologies and supporting engineering decision-making for chromium slag management.

## 1. Introduction

Chromium slag generally refers to a class of chromium-bearing solid wastes characterized by substantial environmental hazards, yet with non-negligible resource potential. In a broad sense, it encompasses chromium-bearing residues generated in the roasting–leaching stages of the chromate industry, commonly termed chromite ore processing residue (COPR) [1,2,3,4]; chromium-containing slags produced by metallurgical processes such as stainless-steel smelting and ferrochromium production [5]; and chromium-bearing sludges and dusts originating from electroplating, chemical manufacturing, and related processes [6,7]. The source term largely governs chromium speciation and host phases. Chromate-industry residues and COPR typically contain a high fraction of hexavalent chromium (Cr(VI)) and chromate-bearing mineral phases, thereby exhibiting pronounced leachability and mobility [8]. In contrast, chromium in metallurgical slags is predominantly present as trivalent chromium (Cr(III)) incorporated into spinel-type mineral phases, which confers relatively higher short-term stability; nevertheless, under certain environmental conditions, re-oxidation may occur and trigger renewed leaching risks [9,10,11,12,13]. Accordingly, chromium slag should not be treated as a single, uniform material system; rather, it represents a “risk–resource” composite formed by multi-source process by-products, and research as well as management should begin with rigorous type identification and scenario-specific classification [14,15,16,17,18,19].

The significance of chromium slag research lies primarily in two dimensions: environmental risk control and resource circularity [20,21]. On the one hand, Cr(VI) is highly toxic and carcinogenic. Under alkaline conditions or specific oxidative environments, it can exhibit high mobility and may enter groundwater, surface water, and soil through leaching, posing long-term exposure risks [22,23,24]. Even after reductive treatment converts Cr(VI) to Cr(III), re-oxidation may still occur in the presence of oxygen, manganese oxides, or during carbonation processes, giving rise to the well-recognized challenge of “short-term compliance but long-term rebound” [25,26,27]. On the other hand, chromium is an essential alloying element with sustained demand in stainless steel and corrosion-resistant alloys, and chromium slags often co-contain valuable components such as Fe, V, and Ni, indicating potential for secondary resource recovery. Promoting a transition from end-of-pipe disposal to safe resource circulation can reduce environmental burdens, decrease reliance on primary resources, and alleviate the pressure of solid-waste stockpiling [28,29,30,31,32].

Overall, existing research and engineering practices have evolved along two parallel pathways [33,34,35,36,37,38,39,40,41,42,43]. One pathway prioritizes risk reduction and regulatory compliance, focusing on technologies such as dry/wet reduction, solidification/stabilization (S/S), and secure landfilling (or controlled storage). Performance evaluation in this stream is typically centered on meeting leaching limits, process implementability, and disposal cost. The other pathway targets resource circularity and value creation, covering bulk utilization in construction materials (e.g., geopolymers, cementitious systems, glass–ceramics, and sintered ceramics), co-treatment within metallurgical process chains (e.g., sintering–blast furnace/electric furnace routes), and the synthesis of functional or catalytic materials. While prior reviews have provided relatively comprehensive summaries of individual technologies or specific application scenarios, the present bottleneck is not the insufficiency of technological options per se. Instead, the most prominent barrier is the lack of systematic evidence that can support cross-route and cross-scenario comparison and inform engineering acceptance decisions. This gap is particularly evident in time-scale evidence for long-term leaching and re-oxidation, in the delineation of boundary conditions governing pollutant burden transfer across solid–liquid–gas media, and in product-side consistency and auditable evaluation systems. These deficiencies collectively prevent many “laboratory-effective” solutions from becoming “engineering-credible”.

Against this backdrop, the novelty of the present review lies not in providing another descriptive inventory of chromium slag treatment technologies, but in proposing a cross-route analytical framework centered on “evidence chain–system boundary–scalable and auditable acceptance.” While existing reviews have mainly focused on summarizing individual detoxification or utilization routes, specific material systems, or single application scenarios, the present work aims to address a more fundamental gap, namely the lack of a unified basis for comparing different technological pathways from the combined perspectives of long-term stability, environmental burden transfer, engineering scale-up, and product-side credibility. In this sense, the review goes beyond a simple compilation of available methods and instead reorganizes them into a mechanism-to-acceptance framework that links chromium speciation and mineral phases, process mechanisms, environmental behavior, risk control, and industrial applicability. At the same time, it seeks to provide a more critical synthesis of the field by identifying the recurring weaknesses that continue to limit cross-study comparability and engineering translation, especially with respect to long-term stability, burden transfer across media, and the gap between laboratory effectiveness and auditable industrial acceptance. Through this perspective, the review aims to clarify why many apparently promising laboratory-scale approaches remain difficult to translate into engineeringly credible solutions, and to identify the key evidence gaps that must be addressed for practical deployment and standardized acceptance.

## 2. Recovery Technologies for Chromium Slag

### 2.1. Pyrometallurgical Recovery and Thermal Treatment: Reduction, Volatilization–Capture, and Atmosphere Regulation

**Figure 1:**
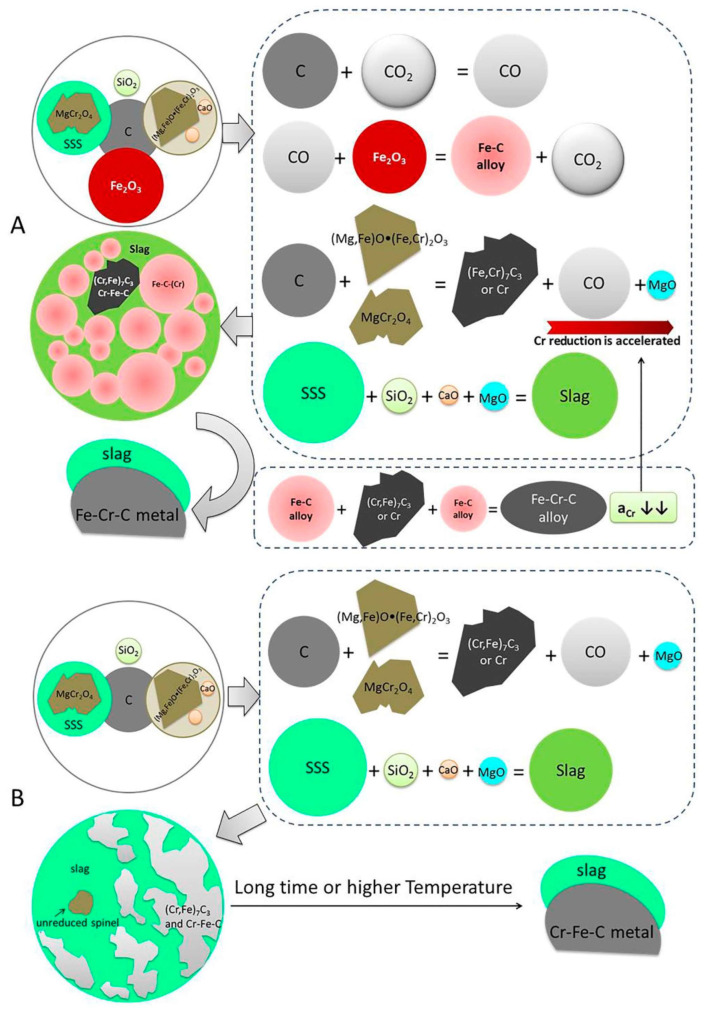
Mechanism of Cr reduction in chromium slag with and without Fe_2_O_3_ ((**A**): in the presence of Fe_2_O_3_, (**B**): in the absence of Fe_2_O_3_) [46].

The fundamental concept of pyrometallurgical recovery is to employ high-temperature conditions to drive chromium valence transformation and phase reconstruction, thereby reducing Cr(VI) to Cr(III), or further to metallic chromium, and achieving resource recovery through melt separation, volatilization–condensation, or alloying with an iron phase [44,45]. Figure 1 illustrates the mechanistic pathways governing chromium recovery from chromium slag via pyrometallurgical treatment [46]. In essence, the process relies on reducing atmospheres generated by carbon and/or CO to convert Cr(VI) to lower-valence species [47]. However, when the objective extends beyond mere regulatory compliance toward resource recovery, the decisive variables are not limited to temperature. Process outcomes are strongly governed by oxygen partial pressure, the CO/CO_2_ ratio, and the presence of additives (e.g., alkaline or sodium salts), which jointly regulate the formation of volatile chromium species and the speciation retained in the solid phase. These factors, in turn, determine whether the process preferentially follows a “volatilization-driven separation and recovery” route or a “solid-phase stabilization and retention” route [48,49].

**Figure 2:**
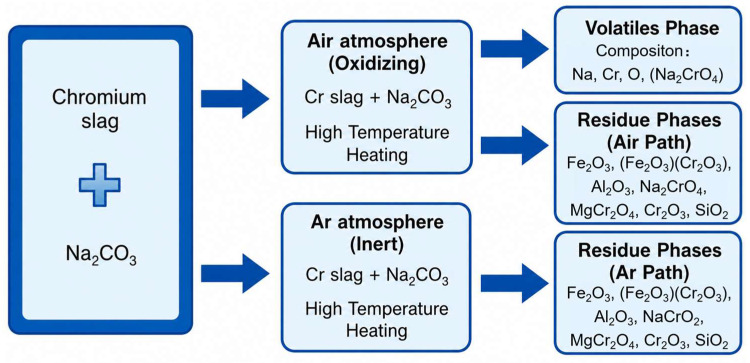
Mechanism of chromium separation formation [49].

Li et al. [49] experimentally demonstrated that atmosphere control at high temperature enables goal switching within the same system. As shown in Figure 2, chromium can be separated by regulating the reaction atmosphere. When chromium slag reacts with additives such as Na_2_CO_3_ under an air atmosphere, chromium-containing compounds that are more volatile or more readily separable can be generated, thereby promoting chromium recovery. In contrast, under an inert atmosphere (e.g., Ar), chromium volatilization can be effectively suppressed, allowing chromium to be retained in the residue predominantly as the less toxic Cr(III) form. These findings indicate that pyrometallurgical processes should be designed through a coupled “atmosphere window–phase evolution” approach to enable synergistic optimization of detoxification, resource recovery, and residue safety, rather than being simplified as a single high-temperature treatment step.

It is important to emphasize that the environmental boundary of pyrometallurgical recovery is often governed by the flue-gas and dust handling stages. Volatile chromium species and chromium-bearing fine particulates may enter the off-gas system; if capture and subsequent management are inadequate, the pollutant burden can be transferred across media rather than eliminated. Therefore, beyond recovery yield and reduction efficiency, pyrometallurgical studies should be supported by whole-process mass-balance analysis to quantify the partitioning of chromium among the metal, slag, dust, and gas phases, and to incorporate capture efficiency and the final fate of collected dust/secondary residues into an integrated assessment.

### 2.2. Hydrometallurgical Recovery: Leaching–Separation–Reduction and Process Intensification

Hydrometallurgical recovery is fundamentally governed by solution chemistry and separation operations. Chromium is selectively dissolved through acid leaching, alkaline leaching, or oxidative/reductive leaching, followed by downstream separation and recovery via reductive precipitation, solvent extraction, ion exchange, or membrane-based processes [50]. Although hydrometallurgical routes are often designed to enhance chromium selectivity, co-leaching of other metals may still occur in complex chromium-bearing wastes and can become a significant constraint on downstream purification and recovery [51,52]. This route offers relatively mild operating conditions, a high potential for selectivity, and convenient integration with multi-element separation schemes. Nevertheless, it is often constrained by high reagent consumption, elevated salt loads and the associated wastewater-treatment burden, as well as the complexity of managing secondary risks arising from both the leaching residues and the spent mother liquor [53].

**Figure 3:**
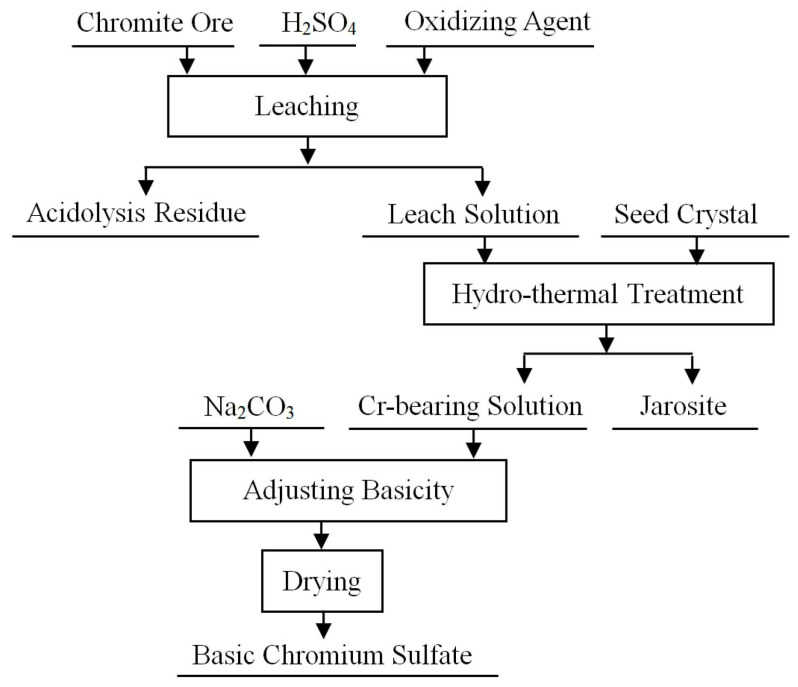
Representative hydrometallurgical route for chromium recovery from chromite ore [54].

Zhang et al. [54] summarized the development of a clean hydrometallurgical process for chromite and proposed a representative chromium-salt production route based on sulfuric acid treatment, as shown in Figure 3. In this route, chromium is extracted into the acidic solution mainly in the form of Cr(III), followed by solid–liquid separation, impurity removal, solution adjustment, and chromium recovery. This process avoids the intentional oxidation of Cr(III) to Cr(VI) required in conventional alkaline processes, and therefore provides a useful reference for designing cleaner hydrometallurgical recovery routes for chromium-bearing materials. Given the inherent characteristics of hydrometallurgical recovery, a process-engineering perspective suggests that process intensification should be treated as a central theme. Intensification units such as electrochemical/electric-field assistance, ultrasonication, microwave irradiation, plasma treatment, and supercritical fluids can be integrated into leaching systems to enhance mass transfer and reaction kinetics, reduce process resistances, and improve selectivity [55]. Accordingly, optimization of hydrometallurgical routes should shift from empirical “recipe trial-and-error” toward modular unit-operation design, with systematic efforts directed at reducing reagent consumption and salt loads, enabling closed-loop circulation, and improving selectivity and scalability [56].

It should be noted that a critical risk associated with hydrometallurgical routes is the transfer of pollutant burdens from the solid phase to the liquid phase. If downstream separation and reduction are incomplete, or if by-products lack a stable and defensible management pathway, the overall environmental burden may be amplified rather than mitigated. Therefore, hydrometallurgical systems should establish a two-sided evidence chain that demonstrates both solution-side compliance and the long-term stability of the solid residues, while incorporating reagent recycling, salt-load control, and by-product fate into a system-level assessment [57].

It should also be recognized that the environmental cost of hydrometallurgical treatment is not fully reflected by chromium removal efficiency alone. Although this route can reduce the direct hazard of the solid phase, it may simultaneously increase reagent consumption, wastewater salinity, neutralization sludge, and the burden of spent-liquor management. Therefore, process assessment should extend beyond detoxification performance and include system-level indicators such as reagent and water consumption, energy demand, wastewater volume and salt load, sludge generation, and residual effluent toxicity. Only within a whole-process mass-balance and life-cycle-oriented framework can the hidden environmental burden of hydrometallurgical routes be compared more fairly with pyrometallurgical and biological alternatives.

### 2.3. Solidification/Stabilization and Mineral-Phase Fixation as a Key Pretreatment for Safe Recovery and Utilization

**Figure 4:**
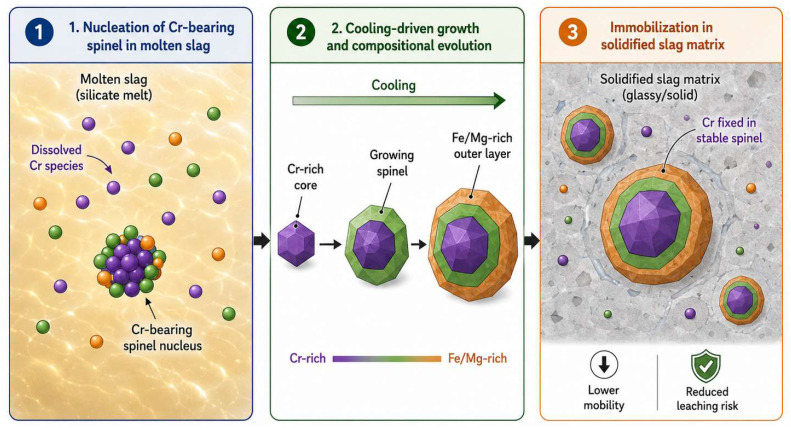
Conceptual schematic of cooling-driven Cr-bearing spinel formation and chromium immobilization in a solidified slag matrix.

Strictly speaking, solidification/stabilization (S/S) is more commonly classified as a disposal or utilization technology [58,59,60,61,62,63,64,65]. In chromium slag systems, however, stabilization frequently serves as a critical pretreatment that enables subsequent recovery or resource utilization. By combining deep reduction with mineralization-based immobilization, Cr(VI) can be transformed and fixed within more stable mineral phases or structural frameworks, thereby reducing the risks of re-oxidation and leaching before the material is directed to construction-material applications or metallurgical process chains [66,67]. As schematically summarized in Figure 4, high-temperature mineral-phase fixation can involve the dissolution of Cr species in molten slag, the cooling-driven nucleation and growth of Cr-bearing spinel, and the final immobilization of chromium within a solidified slag matrix. Previous studies have shown that chromium-bearing phases formed during stabilization are strongly composition-dependent, and that the immobilization behavior of chromium is closely related to phase evolution, matrix structure, and long-term leaching performance [68,69,70]. In addition, research on the solidification of chromium-bearing sludge in China has further established an integrated chain linking “solidified-body strength–multi-scenario durability–human health risk assessment,” and has highlighted the need to supplement key engineering indices such as permeability and radioactivity, as well as to verify adaptability across sludges from different sources prior to field application [71].

Inorganic material-based stabilization of chromium-bearing solid wastes should be supported by systematic datasets that jointly address “maximum incorporation ratio–immobilization capacity–long-term leaching,” so as to accommodate the impacts of source-dependent chromium speciation on stabilization mechanisms and environmental risks. At present, insufficient evidence on long-term leaching behavior and the lack of robust safety-assessment mechanisms remain major bottlenecks that constrain the broader deployment of such resource-utilization routes. Therefore, stabilization should not be treated merely as an end-of-pipe disposal step detached from recovery. Instead, it should be integrated into the full-chain system design for waste recovery and resource utilization, thereby clarifying the feasibility and sustainability of downstream process pathways under controlled risk conditions.

It should also be noted that the predictive capability of current leaching kinetic models for Cr-bearing cementitious or geopolymer matrices remains limited as immobilized chromium may undergo redox transformation during service. Diffusion-based approaches, including Fick-type descriptions, can serve as useful first-order tools for monolithic materials under diffusion-controlled release conditions. However, their direct extrapolation to long-term field performance remains uncertain once carbonation, pH evolution, wetting–drying, freeze–thaw damage, or exposure to external oxidants alters pore structure, chromium speciation, and release behavior. In this sense, short-term standardized leaching tests are valuable for preliminary screening, but they are not sufficient on their own to represent the long-term environmental behavior of Cr-bearing matrices. Before industrial-scale application, a more credible validation framework should therefore combine conventional batch leaching and pH-dependent leaching with semi-dynamic tank leaching for monolithic products, and further incorporate accelerated aging under carbonation, wetting–drying, freeze–thaw, and acid-attack conditions, followed by sequential leaching assessment. Such an approach would help establish a more complete evidence chain linking mineral fixation, redox stability, matrix durability, and long-term release risk.

### 2.4. Bioelectrochemical Coupling: Deep Removal and Remediation of “Inaccessible” Cr(VI)

**Figure 5:**
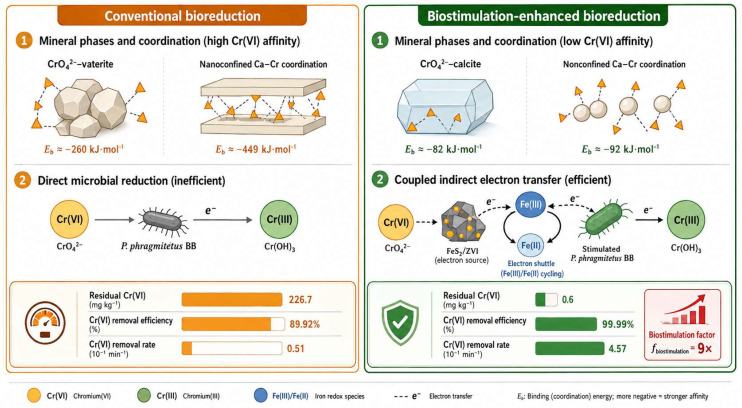
Biostimulation-enhanced microbial detoxification of chromium slag-contaminated soil.

**Figure 6:**
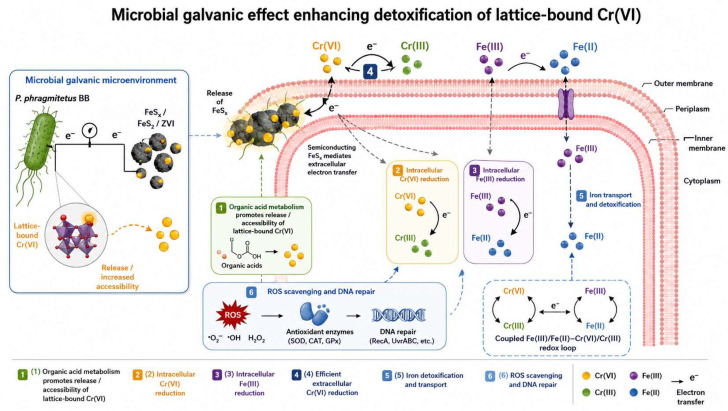
Schematic diagram of the molecular mechanism of the microbial galvanic effect to enhance microbial detoxification of lattice-state Cr(VI).

When Cr(VI) occurs in lattice-bound forms or is encapsulated within host mineral phases, conventional chemical reduction or wash–leaching is often unable to achieve complete removal, typically manifesting as performance plateaus and persistent residual fractions. Li et al. [72] proposed a biostimulation system (e.g., FeS_2_/ZVI) that enhances Cr(VI) accessibility and enables efficient removal by inducing host-phase transformation and strengthening electron-transfer processes. They further constructed a multi-scale mechanistic evidence chain by integrating density functional theory (DFT) with mineralogical and process-level analyses, as shown in Figure 5. Building on this line of work, Li Qi [73] introduced the concept of a “microbial galvanic effect,” in which semiconducting FeSx and electroactive bacteria synergistically enhance extracellular electron transfer, drive the transformation of key mineral phases, and achieve tonne-scale pilot verification. These results suggest tangible engineering potential for this approach, and the underlying principle is illustrated in Figure 6.

This class of technologies broadens the scope of “recovery/management.” Rather than directly recovering metallic chromium, it addresses difficult-to-treat forms such as lattice-bound Cr(VI) through an integrated “release–transformation–stabilization” chain, thereby creating enabling conditions for downstream material utilization or site redevelopment [63]. At the same time, large-scale implementation still depends on robust process control, demonstrated long-term stability, and the establishment of standardized acceptance systems. This requires translating mechanistic parameters—such as electron flux, mineral-phase transformation rates, and microbial community responses—into actionable process-control indicators, and verifying the absence of rebound through long-term monitoring.

### 2.5. Comparative Overview of Chromium Slag Recovery and Management Technologies

To compare the applicability boundaries and engineering potential of different chromium slag recovery and management technologies within a unified evaluation framework, and to address the inherent trade-offs among technical feasibility, environmental risk, and scale-up constraints, it is necessary to conduct a cross-route synthesis from the perspectives of process mechanisms, target waste types, key risks, and technological maturity. Existing studies indicate that pyrometallurgical routes offer clear advantages in processing throughput and scale-up, but require stringent control of high-temperature volatilization/transport and off-gas–dust management. Hydrometallurgical routes provide greater flexibility for selective leaching and multi-element separation, yet are often constrained by high reagent consumption, salt loads, and the risk of shifting pollutant burdens into the liquid phase. Solidification/stabilization and material utilization routes can enable bulk consumption and integration with construction-material value chains, but their deployment is highly dependent on evidence chains for long-term leaching performance and durability. Bioelectrochemical coupling routes show promise for deep transformation of “inaccessible” or lattice-bound Cr(VI); however, field controllability and standardized acceptance still require further verification. On this basis, Table 1 summarizes and compares pyrometallurgical, hydrometallurgical, stabilization/solidification, and bioelectrochemical routes across five dimensions—advantages, limitations, applicable waste types, key risk points, and maturity—to support subsequent process selection, integrated optimization, and evidence-chain development.

## 3. Utilization Pathways of Chromium Slag

### 3.1. Metallurgical Process Co-Treatment: Scale-Up Rationale of the Sintering–Blast Furnace (CAP) Route

**Figure 7:**
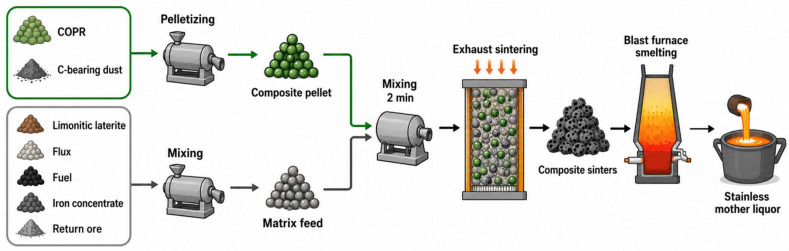
Process flow for blast furnace co-treatment of multi-source chromium slag.

In scenarios where high-throughput management is required, integrating chromium slag into iron and steel process chains for co-treatment has been widely regarded as a promising pathway for industrial-scale deployment. Figure 7 presents a blast furnace-based process for co-treating multi-source chromium slags. Tu Yikang [74] proposed a “sintering–blast furnace co-treatment of multi-source chromium slag and organic solid wastes” route, in which composite agglomeration (CAP) was adopted to improve granulation and bed permeability. The intrinsic reducing environment within the composite pellets enabled deep reduction of Cr(VI), while the encapsulating pellet structure suppressed re-oxidation during the suction cooling stage; a roadmap for industrial implementation was also discussed.

Tu et al. [75] further developed the CAP–blast furnace concept, emphasizing both the mechanistic basis for complete Cr(VI) reduction and the partitioning of recovered chromium within the blast furnace system and stainless-steel melt. Their results indicate that co-treatment of 20% chromium slag with 5% carbon-bearing dust can be achieved without compromising sinter quality. Cr(VI) was fully reduced to Cr(III) and/or metallic chromium, with 12.83% of total chromium present as metallic Cr. The remaining chromium was retained as Cr(III) hosted primarily in spinel phases, such as (Mg,Fe)(Cr,Al)_2_O_4_, or in Cr(III)-bearing calcium–aluminosilicate matrices. After blast furnace smelting, 90.22% of chromium in the sinter entered the stainless-steel melt, enabling closed-loop recycling within the metallurgical system.

In stainless-steel slags, chromium is commonly present in spinel-type phases that are difficult to reduce [65,76,77,78,79,80,81,82,83,84,85,86,87,88,89], which leads to high energy demand for smelting reduction. Physical separation typically yields limited recovery, whereas thermal plasma routes are constrained by equipment availability and cost. Consequently, integrated process combinations are increasingly advocated, and the reutilization of post-recovery residues is considered an essential component of system-level optimization. This perspective is consistent with the closed-loop objective emphasized in Chinese engineering practice, namely “co-treatment–resource recovery–residue management”.

The industrial boundary conditions of metallurgical co-treatment routes are ultimately governed by the predictability and auditable acceptability of system behavior. It is necessary to demonstrate that fluctuations in the composition of chromium slags from different sources [90,91,92,93] do not cause significant deterioration in sinter quality or blast furnace operational stability. In addition, robust strategies must be provided to track and control chromium migration within the dust/off-gas system, so as to avoid transferring pollutant burdens into the fume and dust streams. Moreover, co-treatment of organic solid wastes may introduce S, Cl, and N into the process, imposing additional constraints on off-gas purification and emission control. Therefore, “scalability” depends not only on processing capacity but also on the completeness of whole-process mass balance, clearly defined emission boundaries, and well-established acceptance and auditing systems.

At present, however, the operating windows for major oxides such as SiO_2_, Al_2_O_3_, CaO, and MgO cannot yet be generalized as universal tolerance ranges for all chromium slag co-processing scenarios, because they depend strongly on feedstock source, chromium occurrence state, basicity control, alkali input, and the coupling between sinter quality and blast furnace stability. What can be stated with greater confidence is that compositional variability must be managed within a process window defined jointly by reduction efficiency, phase evolution, slag fluidity, agglomerate quality, and the migration of chromium among metal, slag, dust, and gas phases. In this context, computational thermodynamics provides an important predictive tool. CALPHAD-based platforms such as FactSage are widely used to predict multicomponent and multiphase equilibria, phase relations, and impurity partitioning in pyrometallurgical systems, and they are therefore valuable for pre-screening composition ranges and identifying thermodynamically favorable operating domains. Nevertheless, such equilibrium-based calculations should be regarded as a basis for process design rather than a substitute for pilot-scale or industrial validation, particularly when dust circulation, gas–solid reactions, kinetic constraints, and feedstock heterogeneity become important. Future scale-up studies should therefore combine thermodynamic simulation with mass-balance analysis and continuous-operation data in order to establish more defensible operating windows for CAP–blast furnace co-treatment.

### 3.2. Construction-Material Utilization and Inorganic Products: From Bulk Consumption to Product Credibility

**Figure 8:**
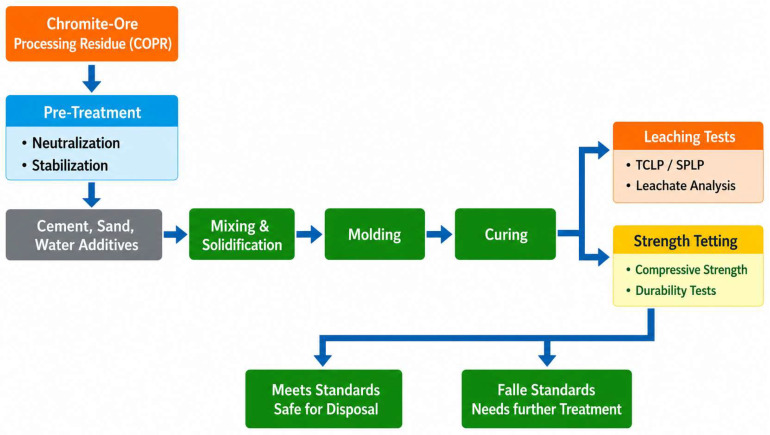
Schematic process flow of cement-based S/S for the utilization of chromium slag.

Construction-material utilization is among the most extensively discussed resource-utilization pathways in China, encompassing a wide range of options such as cement clinker and supplementary cementitious materials, concrete aggregates, bricks, ceramsite, road-base materials, glass–ceramics, cast stone, and sintered ceramics [94,95,96,97]. Figure 8 presents the process flow of cement-based solidification for consuming chromium slag. Chinese reviews have pointed out that some conventional routes have been constrained by policy shifts and industrial restructuring—for example, vertical-kiln cement processes have been phased out under capacity-reduction policies—or by process conditions such as oxidizing atmospheres that lead to incomplete reduction and limited allowable incorporation ratios [98,99,100,101,102,103,104,105,106,107]. Accordingly, pathway optimization and process redesign should be pursued on the premise of clearly defined boundary conditions. Nevertheless, a substantial portion of published work remains at the laboratory scale. One of the major barriers to wider deployment is the lack of robust mechanisms for long-term leaching evaluation and safety assessment, which has resulted in persistent concerns within the industry regarding the long-term credibility of end products.

Inorganic material-based consumption and stabilization can be systematically grouped into four major systems: glass–ceramics, sintered ceramics, cementitious systems, and geopolymers. Source-dependent differences in chromium host phases can markedly influence immobilization mechanisms and leaching risks. It is therefore recommended to establish systematic databases that simultaneously capture immobilization capacity and maximum incorporation ratio, and to conduct long-term leaching investigations under diverse environmental conditions to support large-scale deployment. Research on solidification has further emphasized kinetic modeling and applicability boundaries, suggesting that models should be validated in more complex matrices containing additional heavy metals, and that the effects of different supplementary materials on heavy-metal release should be evaluated.

Overall, the central tension in construction-material utilization lies in the fact that larger consumption volumes entail more stringent requirements for long-term safety evidence. Reliance on short-term leaching compliance and satisfactory mechanical strength alone is generally insufficient to substantiate low-risk claims under variable service environments involving acid rain, salt attack, freeze–thaw cycling, and carbonation. For industrial-scale, bulk construction-material utilization, long-term leaching performance, durability evidence, and standardized quality control should be treated as core requirements in order to establish a product-side evidence chain that is both regulatable and auditable.

### 3.3. High-Value Utilization: Waste-Derived Catalysts/Functional Materials and the Thresholds for Industrial Feasibility

Compared with bulk construction-material utilization, high-value utilization aims to generate higher added value by producing catalysts or functional materials. At the same time, it imposes more stringent requirements on feedstock purity, structural controllability, and clearly defined safety boundaries. Domestic studies have proposed hydrothermal detoxification coupled with crystal-phase reconstruction to convert chromate-industry residues or COPR into low-chromium catalytic materials for CO_2_ hydrogenation or hydrocarbon dehydrogenation. These studies also highlight the need to address potential emissions associated with alkaline agents used in hydrothermal systems and to deepen mechanistic understanding of “high activity at low chromium loading” [108]. In addition, mesoporous Cr/Al_2_O_3_ catalysts prepared using chromium slag as the Cr source have been explored for propane dehydrogenation, with the explicit recommendation that advanced characterization techniques and theoretical calculations should be combined to elucidate active sites and reaction pathways, thereby enabling controllable synthesis and predictable performance [109].

A representative example of “synergistic co-treatment and valorization” is the use of waste chromium slag as a catalyst for the pyrolysis of waste tires, which improves product distribution while promoting Cr(VI) reduction, illustrating the concept of multi-waste synergistic conversion [110]. It should also be recognized that source-dependent differences in chromium speciation can substantially influence stabilization behavior and risk profiles. High-value utilization pathways therefore need to incorporate long-term leaching assessments and end-of-life management evaluations to avoid creating new forms of risk transfer.

A further issue that deserves greater attention in high-value utilization is the role of impurity elements inherently associated with chromium slag, such as Fe, Ni, V, and Si. Depending on their occurrence state and distribution, these species may modify the dispersion of chromium, alter surface acidity/basicity and redox behavior, block or dilute catalytically active sites, or in some cases, introduce additional functionalities that change activity and selectivity. For this reason, the performance of slag-derived catalysts should not be interpreted solely in terms of nominal chromium content. More reliable understanding requires the combined use of advanced characterization methods capable of distinguishing chromium environments from impurity-related species, including surface-sensitive spectroscopy and local-structure analysis, together with adsorption- or probe-based evaluation of accessible active sites. From an engineering perspective, these high-resolution methods may not be directly transferable to routine plant control, but they can help establish correlations between catalytic performance and simpler quality indices, thereby providing a basis for industrial quality control of slag-derived functional materials.

Accordingly, the key threshold for industrializing high-value utilization lies in establishing a robust mapping from “feedstock variability–synthesis window–structural/active sites–performance–safety,” while completing environmental boundary evaluations across the full life cycle, including material service, regeneration, and disposal stages. Such evidence is essential to ensure that the overall benefits outweigh the associated risks and costs.

### 3.4. Multi-Element Synergistic Recovery and Circular Utilization: Coupling with Co-Occurring Resources Such as V/Fe/Ni

Chromium slag and related metallurgical solid wastes often co-contain elements such as V, Fe, and Ni. The coupling among multiple valuable elements has stimulated research on synergistic recovery and circular utilization. A representative domestic study investigated calcification roasting followed by acid leaching of vanadium–chromium slag, identifying a separation window under specific conditions where high vanadium leaching can be achieved while chromium dissolution remains minimal. This provides a process basis for cleaner vanadium extraction and for mitigating chromium-migration risks [111]. Starting from Cr/V-bearing industrial solid wastes, another line of work has systematically summarized hydrometallurgical routes and process-intensification strategies for metal removal and separation, emphasizing the spectrum of intensifiable units and the evolving technology portfolio [51]. Meanwhile, for stainless-steel slags, the reutilization of post-recovery residues should be advanced in parallel with chromium recovery; otherwise, the system may continue to bear substantial solid-waste burdens and associated environmental risks.

These observations indicate that multi-element recovery should not be evaluated solely by maximizing the recovery yield of a single target element. Instead, system-level optimization is required to jointly address “recovery performance–residue fate–long-term safety–economic feasibility,” while selecting the most appropriate closed-loop pathway in line with industrial value-chain demands.

### 3.5. Summary of Typical Industrial Routes and Evidence-Gap Assessment

From the perspective of industrial deployment and large-scale consumption, current chromium slag resource-utilization routes with the greatest scale potential can be broadly categorized into three types: metallurgical process co-treatment (CAP–sintering–blast furnace), construction-material utilization (bulk consumption in inorganic materials), and product-oriented solidification (engineered products such as solidified bodies and unfired bricks). These routes differ in processing scales, modes of resource recovery, focal points of environmental risk control, and industrialization thresholds. The first two routes primarily emphasize bulk consumption and integration with existing industrial value chains, whereas the third route aligns more closely with an engineering acceptance logic that links the “treatment–product–application scenario.” To further distill the technical characteristics of these routes and compare their practical constraints during industrial implementation, this review synthesizes the major advantages, limitations, and maturity levels of each pathway, as summarized in Table 2.

The construction-material utilization route enables bulk consumption through established industrial value chains such as cement, geopolymers, glass–ceramics, and sintered ceramics, yet its dissemination is fundamentally constrained by insufficient long-term safety evidence. The lack of robust mechanisms for long-term leaching evaluation and safety assessment has led to persistent concerns within industry regarding product credibility over extended service periods. To address this gap, systematic databases and long-term leaching studies under diverse environmental conditions are required so that maximum incorporation ratios can be optimized in tandem with immobilization capacity. Accordingly, the most critical evidence gaps for this route lie in cross-scenario durability and long-term leaching performance. Compliance demonstrated solely by short-term leaching tests and satisfactory mechanical strength is generally inadequate to substantiate low-risk claims under evolving service environments, including acid rain exposure, salt attack, freeze–thaw cycling, and carbonation.

Product-oriented solidification is more closely aligned with engineering acceptance logic because it naturally forms a closed loop linking “treatment–product–scenario-specific risk.” Studies integrating chromium-bearing sludge solidification, stability testing under extreme environmental conditions, and RBCA-based human health risk assessment have further highlighted the need to supplement key engineering indices such as permeability and radioactivity, and to verify adaptability across wastes from different sources. In parallel, work emphasizing leaching-kinetics modeling and applicability boundaries have suggested that models should be validated in multi-metal systems and that formulation effects on release behavior should be systematically evaluated. For this route, evidence gaps are primarily associated with transferability and standardization. Given the pronounced heterogeneity among waste sources, the absence of a unified indicator matrix and clearly defined key quality control points make it difficult to translate case-specific successes into reproducible industry norms.

Overall, the three industrialization pathways exhibit a common pattern. The closer a route is to large-scale bulk consumption—particularly metallurgical co-treatment and construction-material utilization—the more essential it becomes to move beyond “single-point compliance” toward comprehensive substantiation based on clearly defined system boundaries, long-term evidence chains, and standardized indicators. Although product-oriented solidification more readily supports scenario-based acceptance and auditing, it likewise requires transferable models across waste sources and a coherent standard system to enable broad, scalable deployment.

## 4. Current Challenges and Development Trends (Novel Stabilization, Green Innovations, and Scale-Up Barriers)

### 4.1. Long-Term Stabilization of Cr(VI): Re-Oxidation, Slow Release, and Multi-Scenario Durability

One of the central challenges in chromium slag management is that Cr(VI) stabilization is not merely a matter of instantaneous reduction. Rather, it is a system-level problem of maintaining low valence states and low mobility over extended time scales [112]. The lack of robust mechanisms for long-term leaching–safety assessment continues to constrain broader deployment, and future priorities increasingly emphasize database construction and long-term leaching investigations under diverse environmental conditions [113]. In contaminated-soil systems, the importance of host-phase limitations and mineral-phase transformation has become particularly evident. Evidence from approaches integrating microbial galvanic effects with mineral-phase transformation and long-term monitoring suggests that a coupled “phase transformation–electron transfer–long-term stability” pathway may be critical for addressing difficult-to-treat forms such as lattice-bound Cr(VI).

Accordingly, future stabilization strategies are expected to shift from simply strengthening reductants toward multi-mechanism synergy, including mineral-phase engineering (e.g., spinel formation, layered double hydroxides, and aluminosilicate network fixation), structural encapsulation, and environmental buffering. Long-term credibility will need to be established through durability testing across multiple scenarios rather than relying on short-term indicators alone.

Further, it should be recognized that the long-term stability of immobilized Cr cannot be discussed without considering the pathways by which apparently stable Cr(III) may be re-oxidized under service or disposal conditions. Among the mechanisms that have been most widely discussed, particular attention has been given to oxidation promoted by manganese oxides, carbonation-induced changes in pore solution chemistry, and other redox-sensitive environmental factors that may alter chromium speciation and remobilization behavior. These observations indicate that conventional leaching tests, although useful for initial screening, do not adequately represent the coupled effects of chemical aging and redox transformation. Future validation frameworks should therefore incorporate accelerated exposure conditions that are more closely related to realistic service environments, such as carbonation, wetting–drying, humidification–drying cycles, freeze–thaw damage, and alternating oxidizing and reducing atmospheres, followed by leaching and speciation assessment. In this way, durability evaluation would move beyond simple compliance testing and better reflect the long-term re-oxidation risk that ultimately governs engineering credibility.

### 4.2. Green Utilization and “Waste-to-Treat-Waste”: From Concepts to Verifiable Systems

Recent work on reductant optimization and “waste-to-treat-waste” concepts—together with biomass-based reduction in hydrometallurgical systems and dual-waste synergistic pyrolysis—have been complemented by engineering practices that exploit by-product waste streams to enhance reduction and lower cost [114,115]. Collectively, these efforts form a portfolio of “waste-stream coupling” innovations, in which external waste streams are repurposed as reductants, catalysts, or process media to reduce primary resource inputs and potentially improve overall environmental performance.

Nevertheless, green innovations must demonstrate system-level superiority through rigorous assessment. For example, synergistic thermal conversion may introduce organic pollutants, sulfur-/chlorine-containing off-gases, or challenges associated with char and residue disposal. Similarly, using waste-derived reductants in hydrometallurgical processes can introduce complex ions, increasing salt loads and downstream treatment burdens. Without whole-process mass balance, well-defined emission boundaries, and long-term risk evaluation, such innovations are unlikely to overcome barriers to large-scale deployment.

### 4.3. Cost and Large-Scale Deployment: Process Windows, Feedstock Variability, and Standardized Acceptance

The common constraints on scale-up arise from cost, throughput requirements, and uncertainty. Metallurgical co-treatment routes offer high capacity but must address the impacts of multi-source slag compositional variability on sintering/blast furnace stability [74], product quality, and emissions, while establishing auditable control and acceptance schemes for chromium migration within dust/off-gas systems. Construction-material utilization and product-oriented solidification must simultaneously satisfy leaching compliance and engineering performance indices such as strength and durability, and must secure a regulatable and auditable evidence chain through long-term leaching and risk assessment [116].

These issues collectively point to the need for an upgraded evaluation paradigm. The call for database-driven approaches and the recognition of inadequate long-term leaching assessment converge on a clear trend: evaluation should expand from single metrics to a multidimensional indicator system. Such a system should jointly consider safety (short- and long-term leaching and re-oxidation), resource-utilization performance (recovery yield, consumption capacity, and value added), environmental performance (wastewater salt loads, off-gas/dust migration, and secondary residues), economic feasibility (reagent/energy consumption and capital investment), engineering implementability (process-window width and sensitivity to feedstock variability), and compliance/acceptance (standards and product regulation).

It should also be recognized that the economic performance of chromium slag management routes cannot be represented by a single nominal treatment cost, because the overall cost depends strongly on waste composition, pretreatment requirements, transport distance, plant scale, energy prices, reagent demand, residue-management obligations, and whether value recovery can offset part of the processing burden. For this reason, direct comparison among metallurgical co-treatment, construction-material utilization, solidification, and disposal-oriented options should be made within a consistent system boundary that includes not only capital and operating expenditures, but also costs associated with environmental control, secondary-residue handling, compliance monitoring, and long-term liability. In many cases, secure landfills or controlled storage may appear less expensive in the short term, whereas recovery-oriented pathways may become more competitive when resource value, avoided disposal burden, and policy incentives are taken into account. Therefore, future techno-economic assessments should move beyond isolated processing costs and explicitly consider the interaction between engineering scale, environmental obligations, marketability of products, and regulatory instruments such as landfill taxes, subsidies, or green-procurement incentives.

### 4.4. Trends in Novel Stabilization Methods: Mineral-Phase Engineering, Geopolymers, and Multi-Scale Evidence Chains

One prominent trend in stabilization is the transition from reduction-centered approaches toward mineral-phase engineering and structural fixation [58,60,61]. The relative stability of spinel and aluminosilicate network structures for chromium immobilization has received increasing attention, and long-term leaching studies supported by systematic databases are being recognized as key tasks [117]. Geopolymers and slag-based binders are also gaining traction due to their strong immobilization potential and favorable engineering performance. Recent domestic studies have begun to integrate multi-scenario durability testing and health risk assessment into the evaluation framework, while proposing further improvements to engineering indices.

A parallel trend is the strengthening of mechanistic evidence chains. Studies that integrate density functional theory (DFT) with mineralogical and microbiological evidence, and that extend from mechanistic validation to pilot-scale verification and long-term monitoring, illustrate the ongoing shift from laboratory proof to engineering proof. Going forward, the competitiveness of stabilization innovations will depend not only on formulation advances, but also on whether they can provide reproducible, quantifiable, and scalable multi-scale evidence chains.

### 4.5. Outlook on Research Objectives: From Short-Term Optimization to Long-Term System Building

Taken together, progress in integrated chromium slag treatment should no longer be confined to optimizing individual process parameters. Instead, it should advance simultaneously on two levels: near-term implementable improvements and long-term system building. In the near term, research priorities should focus on clarifying boundary conditions of existing routes, optimizing key control parameters, and strengthening baseline evaluation frameworks. Particular emphasis should be placed on generating comparable and reproducible experimental and pilot-scale datasets related to Cr(VI) re-oxidation risk, leaching behavior, process-window width, and the environmental burdens associated with by-product streams.

In the longer term, chromium slag management and resource utilization will require an application-oriented system framework that moves beyond single-point compliance toward coordinated evaluation of “long-term safety–resource efficiency–environmental performance–economic feasibility–standardized acceptance.” Table 3 summarizes the key research objectives and implementation priorities for chromium slag management and resource utilization from both short- and long-term perspectives. This will entail sustained efforts in database development, standard-system refinement, multi-scenario durability verification, and the implementation of industrial demonstration projects. A further priority for improving comparability is the normalization of a limited set of core testing and reporting parameters before attempting broader methodological unification. For leaching-related studies, greater consistency is especially needed in particle-size range, liquid-to-solid ratio, test duration, final pH, temperature, and the chemical composition or ionic strength of the leaching medium, because variations in these factors can strongly affect the apparent release behavior of chromium and other associated elements. At the same time, greater transparency is needed in reporting waste source, pretreatment history, curing or thermal-treatment conditions, and matrix form, so that results from different studies can be interpreted within a comparable system boundary. In this context, an open database structured according to FAIR principles would be both feasible and valuable, provided that it includes harmonized descriptors for raw-material characteristics, process conditions, product form, and short- and long-term environmental performance. Such a platform would not only improve meta-analysis and cross-study comparison, but also support model development, standard refinement, and more credible engineering decision-making. In other words, future research must not only develop treatment materials or processes that are “more effective,” but also build comprehensive evidence chains and implementation systems that are “more credible, scalable, and regulatable”.

## 5. Conclusions

In summary, research on the detoxification and resource utilization of chromium slag has established a broad portfolio of technological routes, spanning hydrometallurgical processes, pyrometallurgical/thermal treatments, solidification/stabilization and material utilization, bioelectrochemical coupling, and metallurgical co-treatment for bulk consumption. These routes continue to evolve along directions such as waste-to-treat-waste strategies, process intensification, mineral-phase engineering, and high-value functional materials. From an industrialization perspective, metallurgical process co-treatment and construction-material utilization represent the two principal pathways with the greatest scale-up potential. The former leverages existing iron and steel process chains to enable bulk consumption and chromium recovery, whereas the latter relies on inorganic material systems to achieve broad-spectrum consumption; however, the latter must be underpinned by long-term leaching and durability evidence to establish product-side credibility. From a scientific standpoint, long-term stabilization of Cr(VI) remains a cross-cutting bottleneck. Re-oxidation, slow release, and durability across multiple scenarios determine whether short-term compliance can be translated into long-term credibility, and they constitute fundamental constraints on technology deployment and regulatory acceptance.

Accordingly, future research and engineering implementation should not focus on simply expanding the list of process “labels.” Instead, application scenarios should guide the explicit definition of system boundaries across the full chain—inputs, processes, emissions, products, and residue fate. An integrated evidence chain should be established to connect mechanistic research with engineering practice, covering “speciation/mineral phases–process mechanisms–environmental behavior (short- and long-term)–risk assessment–engineering scale-up–standardized acceptance.” In parallel, database development and a unified indicator matrix are needed to support cross-source transferability and large-scale decision-making. On this basis, chromium slag management can move beyond end-of-pipe control and achieve a genuine paradigm shift from high-risk solid waste toward a controllable secondary resource.

## Figures and Tables

**Table 1:** Comparative overview of major technology routes for chromium slag recovery and management.

Technology Route	Core Principle and Representative Practices	Advantages	Limitations	Applicable Waste Types	Key Risk Points	Maturity (Engineering Readiness)
Pyrometallurgical routes (thermal treatment/smelting reduction/atmosphere control)	High-temperature reduction of Cr(VI) → Cr(III) and/or metallic Cr; regulation of volatilization-based recovery or solid-phase immobilization via atmosphere (air/inert) and additives	High throughput; relatively simple process flows; low wastewater burden; readily coupled with kilns and metallurgical equipment	High energy demand; volatilization and dust-borne transport require strengthened off-gas control; feedstock variability narrows the process window; comparatively insufficient evidence for long-term stability	Dry detoxification of high-Cr(VI) chromium slag; pretreatment/recovery of chromium-bearing metallurgical slags; synergistic thermal conversion systems	Volatile Cr species and Cr-bearing dust entering off-gas streams; re-oxidation due to improper cooling; pressure of secondary dust/slag management	High (detoxification/disposal)/medium (high-purity recovery): dry detoxification is relatively mature; refined atmosphere-controlled recovery and whole-process control still require engineering verification
Hydrometallurgical routes (leaching–separation–reduction/precipitation)	Acid/alkali/oxidative leaching; solution-side reductive precipitation or extraction-based separation; optional integration of process-intensification units (electrochemical assistance/ultrasonication/microwave, etc.)	Mild operating conditions; high potential for selectivity; suitable for multi-element separation and refined recovery; can be coupled with green reductants and “waste-to-treat-waste” concepts	High reagent consumption and salt loads; complex wastewater/mother-liquor treatment; risk of burden transfer from solid to liquid phase; difficulty in closed-loop circulation	COPR/chromate-industry residues; multi-element recovery systems (e.g., Cr–V)	Discharge risk due to incomplete solution-side reduction; salt accumulation and by-product disposal; long-term safety of leaching residues	Medium: abundant laboratory and pilot studies, but large-scale deployment is constrained by cost, salt load, and insufficient closed-loop evidence
Stabilization/solidification and material utilization (S/S, geopolymers, cementitious systems, glass–ceramics, etc.)	Reduction/mineralization plus structural encapsulation: immobilization of Cr via spinel formation, aluminosilicate networks, or hydration products; products used as construction materials or disposed safely	Enables bulk consumption and integration into construction-material value chains; potential to balance engineering performance and risk control	Uncertainty in long-term leaching and re-oxidation; lack of standardized acceptance criteria; insufficient product consistency and transferability across sources	Low-/medium-Cr wastes and treated residues; material utilization of chromium-bearing sludge, dust, and some metallurgical slags	Increased leaching under changing service environments; re-oxidation; product quality fluctuations	Medium–high (solidification for disposal)/medium (resource-utilization products): disposal-oriented solidification is relatively mature; construction-material utilization still requires standardization and long-term databases

**Table 2:** Comparative overview of major chromium slag resource-utilization pathways.

Pathway Type	Representative Process(es)	Key Advantages	Key Limitations	Technology Maturity
Metallurgical process co-treatment	CAP–sintering–blast furnace co-treatment	Leverages existing iron and steel process chains with large processing capacity and strong continuity; enables deep reduction of chromium in slag and allows part of chromium to be recovered into hot metal or the melt system; offers synergistic benefits in waste minimization, detoxification, and resource utilization, with high potential industrial throughput.	Sensitive to feedstock compositional variability; must ensure sinter quality and blast furnace operational stability; stringent requirements for controlling chromium migration/partitioning among slag, metal, dust, and gas phases and for off-gas purification; co-treatment with organic solid wastes may introduce impurities such as S, Cl, and N, increasing the difficulty of emission control.	Relatively high. Clear directions for industrial scale-up and available process infrastructure exist; however, whole-process mass balance, emission boundaries, and cross-source adaptability still require further verification.
Bulk construction-material utilization	Cement clinker/supplementary cementitious materials, concrete aggregates, bricks, ceramsite, glass–ceramics, sintered ceramics, etc.	High consumption capacity with broad application scenarios; readily integrated into existing construction-material value chains; leaching risk can be reduced via solidification, encapsulation, and phase reconstruction; diversified product forms offer strong potential for bulk resource utilization.	Highly influenced by feedstock properties, firing atmosphere, and maximum incorporation ratios; some routes risk incomplete reduction or chromium reactivation; insufficient evidence for long-term leaching, durability, and service-environment safety, leading to persistent industry concerns regarding long-term product credibility.	Moderate to relatively high. Extensive laboratory and pilot studies exist, and some pathways have an industrial basis; however, broad deployment still depends on robust long-term safety evidence.
Product-oriented solidification	Cement-based solidified bodies, unfired bricks, engineered backfill materials, etc.	Relatively simple processes with low equipment requirements; aligns closely with an auditable “treatment–product–application scenario” closed loop, facilitating engineering acceptance; formulation optimization can improve compressive strength, leaching stability, and environmental risk performance.	Product performance is strongly affected by waste-source variability, resulting in limited cross-source transferability; studies are often case-specific and lack unified quality-control indicators and standard systems; further evidence is needed on permeability, durability, and long-term health risk assessment.	Moderate. Process pathways are relatively clear and engineering barriers are comparatively low, but standardization, large-scale deployment, and product credibility systems remain underdeveloped.

**Table 3:** Outlook on research objectives for chromium slag management and resource utilization.

Objective Dimension	Short-Term Objectives	Long-Term Objectives
Cr(VI) stabilization and re-oxidation control	Optimize reduction–solidification conditions, and clarify how pH, Eh, curing conditions, and mineral-phase assemblages affect Cr(VI) reduction and re-oxidation; supplement short-term leaching tests and preliminary durability evaluation.	Establish a long-term stabilization evaluation system covering multiple scenarios (acid rain, salt attack, freeze–thaw cycling, carbonation, etc.) to enable prediction of Cr speciation, re-oxidation behavior, and long-term release risk.
Mineral-phase engineering and novel stabilization materials	Screen representative mineral-phase engineering systems (e.g., spinel, LDHs, aluminosilicate networks, and geopolymer systems), and elucidate their Cr immobilization mechanisms and applicability boundaries.	Develop a stabilization material platform that integrates high immobilization capacity, environmental adaptability, and engineering implementability, thereby promoting a shift from “empirical formulations” to “mechanism-guided design.”
Green utilization and waste-to-treat-waste strategies	Evaluate the feasibility of using by-product waste streams, biomass, and industrial by-products as reductants, supplementary materials, or reaction media, and clarify their impacts on reduction efficiency and pollutant migration.	Establish an integrated mode of “waste-stream coupling–resource substitution–environmental benefits,” and develop green utilization pathways that can be verified through life-cycle assessment (LCA) and system-wide mass-balance analysis.
Environmental risk control	Identify key environmental risk nodes, including wastewater salt loads, off-gas dust, and secondary residues, and supplement data on major contaminant transport pathways.	Establish a whole-process environmental risk control system covering solid, liquid, and gas media, enabling systematic reduction in secondary pollution and full-process regulation/oversight.
Scale-up and process engineering	Characterize compositional variability across chromium slags from different sources and quantify its effects on process windows, reagent consumption, energy demand, and product quality; conduct studies bridging lab-scale to pilot-scale.	Develop engineering process flows and scale-up models applicable to multi-source chromium slags, improving robustness against feedstock variability and stability under continuous operation.
Resource-utilization performance and product-side credibility	On the basis of leaching compliance, supplement product-performance evaluation (e.g., strength, durability, permeability) and clarify the application scenarios suitable for different utilization routes.	Establish a utilization-evaluation framework that jointly considers consumption capacity, value added, long-term safety, and service reliability, promoting a transition from “manufacturable” to “usable and acceptable” products.
Evaluation methods and data support	Harmonize commonly used test methods, evaluation indicators, and data reporting formats to enhance comparability across studies.	Build a database for chromium slag treatment and utilization to enable systematic accumulation and sharing of performance and risk characteristics across processes, materials, and environmental conditions.
Standards, specifications, and regulatory acceptance	Review existing standards for leaching, product performance, and environmental safety evaluation, and clarify gaps between current research outputs and engineering acceptance requirements.	Develop a standards and specification system covering feedstock classification, process control, product quality, long-term safety, and scenario applicability to support industrialization and regulatory implementation.
Mechanistic research and evidence-chain development	Integrate mineralogical characterization, leaching experiments, and fundamental reaction-mechanism studies to establish relationships among “material–structure–performance.”	Develop a multi-scale evidence chain that links microscopic mechanisms, long-term monitoring, and engineering demonstration, thereby improving reproducibility, interpretability, and industrial credibility.

## Data Availability

No new data were created or analyzed in this study. Data sharing is not applicable to this article.
